# Influence of Carbonization Temperature on the Tunable Work Function of Graphite-like Biomass Carbon Derived from *Wolffia globosa* and Its Morphology and Phase Structure Properties

**DOI:** 10.1021/acsomega.6c02592

**Published:** 2026-07-21

**Authors:** Sudarat Khwanmueang, Woranuch Sudthongkong, Akkawat Ruammaitree, Orapan Saensuk, Poramed Wongjom, Yingyot Infahsaeng, Kunthaya Ratchaphonsaenwong, Pantiwa Petpadoong, Wasan Maiaugree

**Affiliations:** † Thammasat University Research Unit in Energy Innovations and Modern Physics (EIMP), 65100 Thammasat University, Pathum Thani 12120, Thailand; ‡ Division of Physics, Faculty of Science and Technology, 546360 Thammasat University, Pathum Thani 12120, Thailand; § NSTDA Characterization and Testing Service Center, 61191 National Science and Technology Development Agency, Pathum Thani 12120, Thailand; ∥ Research and Academic Services Division Faculty of Science, 26684 Khon Kaen University, 123 Mittraparb Road, Nai-Meuang, Meuang, Khon Kaen 40002, Thailand

## Abstract

Graphite electrodes are widely used in electrochemical and energy-related applications; however, their properties are often limited by the natural origin of graphite, restricting tunability. Herein, we present a simple and scalable route to produce graphite-like carbon from *Wolffia globosa* biomass with tunable structural and electronic properties. This rapidly growing aquatic plant, abundant in Southeast Asia, was selected due to its high biomass yield, short cultivation period, and intrinsic nitrogen- and oxygen-containing composition, enabling in situ heteroatom self-doping during carbonization. The biomass was carbonized under an argon atmosphere at 900–1300 °C, producing samples denoted as W900–W1300. The effects of carbonization temperature on the structural, chemical, and electronic properties were systematically investigated. FTIR analysis showed progressive removal of oxygen-containing functional groups above 1100 °C, indicating recovery of sp^2^ carbon domains. Raman spectroscopy revealed a decrease in the I_D_/I_G_ ratio from 1.320 to 1.000 and an increase in crystallite size from 14.56 to 19.22 nm, confirming enhanced graphitization. XPS results further demonstrated an increase in the sp^2^/sp^3^ ratio with increasing temperature. SEM and TEM analyses showed the formation of well-organized nanosheet structures, while BET analysis confirmed enhanced surface area and pore development at higher temperatures. UPS measurements indicated tunable work functions ranging from 2.66 to 4.13 eV. These results demonstrate that *Wolffia globosa* is a promising renewable precursor for producing graphite-like carbon for energy storage and optoelectronic applications.

## Graphical abstract

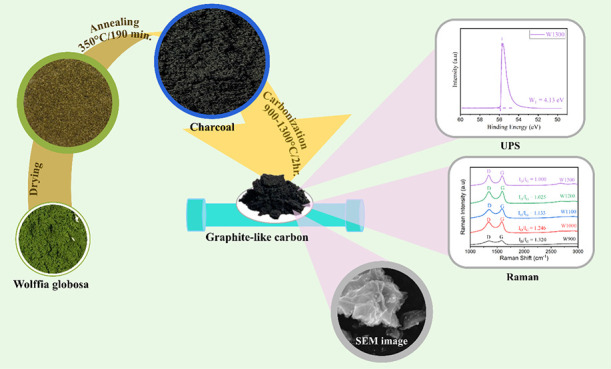

## 1 Introduction

Carbon is an exceptionally versatile element in materials science due to its ability to form various allotropes, ranging from amorphous carbon to highly ordered crystalline structures such as graphite, carbon nanotubes, fullerenes, and graphene. This structural diversity results in distinct physicochemical properties, enabling the design of carbon materials tailored for diverse technological applications. Graphite-like carbon, in particular, is prominent in electrochemical systems owing to its high electrical conductivity, excellent chemical stability, and cost-effectiveness.^1–3^

Graphite has long been utilized as an electrode material in energy conversion and storage technologies, including batteries, supercapacitors, and solar cells.^4^ Its layered structure facilitates efficient charge transport, while its thermal and chemical stability ensure durability under operational conditions. However, natural graphite often exhibits variations in physicochemical properties depending on its origin, purification process, and degree of crystallinity. Such inconsistencies can lead to unstable performance, limiting its use in advanced electronic and energy devices.

To overcome these limitations, current research focuses on biomass-derived graphite-like carbon, which offers greater sustainability and tunable properties. The carbonization process plays a critical role in determining the structural and electronic properties of the resulting material, such as the degree of graphitization, defect density, morphology, porosity, and work function.^5^ These factors directly influence charge mobility and interfacial behavior in electrode systems. Compared to reduced graphene oxide (rGO), which often contains structural defects and an incomplete sp^2^ network, graphite-like carbon synthesized through controlled high-temperature treatment can develop a more ordered sp^2^ structure. This leads to enhanced electrical conductivity and superior thermal and chemical stability.^6^

Numerous studies have explored the conversion of biomass into high-performance carbon materials. For instance, olive oil mill waste has been processed into graphitic carbon for high-capacity gas adsorption of CO_2_, CH_4_, and H_2_ enabled by its large surface area and pore volume.^7^ Coconut shell-derived graphite has been successfully employed as a charge transport material in perovskite solar cells, significantly reducing production costs by replacing gold.^8^ Furthermore, cantaloupe peel graphite, when used in composite electrodes with fly ash for Dye-Sensitized Solar Cells (DSSCs), demonstrated a power conversion efficiency of 5.81%, comparable to platinum electrodes (5.91%).^9^ Oil palm petioles, cassava residue, and residual cannabis biomass were used to synthesize rGO-like carbon as a catalyst material for counter electrodes in DSSCs.^10–12^ Recent developments also include the use of boron as a catalyst to induce graphitization in wood-derived carbon at a relatively low temperature (900 °C), resulting in lithium-ion battery capacities of 505 mAh/g.^13^ Additionally, biocarbon from rice straw, corn cobs, peanut shells, and bamboo carbonized at 1200 °C has been used in perovskite solar cells, with bamboo-derived graphite achieving an efficiency of 12.82%.^5^

For solar cell electrodes and related applications, the work function is a critical parameter for determining energy level alignment and electron transport pathways, such as those in the top electrodes,^14^ interlayers within electron transport layers of perovskite solar cells,^15^ and catalyst electrodes.^16^ Therefore, the capability to tune the work function of carbon materials can enhance device performance and enable the selection of suitable electrodes.^17^ Additionally, other properties such as morphology, phase structure, chemical functional groups, and porosity are important for electrodes and were systematically studied.

*Wolffia globosa*, commonly known as Khai-Nam or “Phum” in Thailand, is widely distributed in tropical and temperate regions, especially in Southeast Asia,^18^ including Thailand, Vietnam, and China, as well as in India, Bangladesh, Myanmar, and Sri Lanka. It is also found in Central and Southern Africa, Madagascar, and Brazil. It is the world’s smallest flowering plant, characterized by a rootless, *Wolffia globosa*, or ovoid frond with a succulent texture. Botanically, it lacks a vascular system and internal air spaces, reproducing primarily through budding. *Wolffia globosa* exhibits high growth rates, ease of cultivation, and a biochemical composition rich in proteins and organic carbon-containing compounds.^19^ The cultivation period for *W. globosa*, from seed release to harvest, ranges from 7 to 14 days, with an average duration of approximately 2 weeks. Therefore, this biomass represents a rapid and efficient route for carbon source production. Notably, its relatively high nitrogen and oxygen content facilitates natural heteroatom doping within the carbon framework during carbonization. This self-doping effect enhances electrical conductivity and electrochemical properties,^20,21^ making *W. globosa*-derived carbon a compelling sustainable alternative to traditional carbon materials.

Developing simple and effective strategies to tune the work function and related properties of biomass-derived carbon is of considerable interest because work function governs electron transfer and energy-level alignment at interfaces. Therefore, it is a key parameter for optimizing the performance of solar cells, batteries, electrochemical energy storage systems, photocatalysis, sensors, OLEDs, and advanced carbon materials. However, the relationship between carbonization conditions and work function in biomass-derived carbon remains poorly understood. In particular, although carbonization temperature is known to influence graphitization and defect formation, its effect on the evolution of electronic properties, especially work function, has not been systematically established. Despite the increasing interest in biomass-derived graphitic carbon, rapidly renewable aquatic biomass remains largely unexplored as a precursor for producing graphite-like carbon materials. Owing to its exceptionally rapid growth, high biomass productivity, low cultivation demand, and naturally abundant nitrogen and oxygen-containing constituents, *Wolffia globosa* represents a promising and sustainable precursor for graphite-like carbon production.

To address these gaps, this study investigates the influence of carbonization temperature on the work function and related properties of biomass-derived carbon derived from *Wolffia globosa*. Graphite-like carbon was synthesized through carbonization at temperatures ranging from 900 to 1300 °C, and the effects of carbonization temperature on graphitization behavior, crystallinity, morphology, porosity, electrical conductivity, and work-function characteristics were systematically investigated. The knowledge obtained from this study provides a simple route for tailoring carbon properties toward specific energy and electronic applications. Overall, this work demonstrates that *Wolffia globosa* is a promising sustainable carbon source for advanced energy and environmental applications.

## 2 Experimental Section

### 2.1 Synthesis of Graphite-like Carbon from *Wolffia globosa*

Graphite-like carbon was prepared from a biomass feedstock, *Wolffia globosa*. The biomass samples were obtained from a natural pond located in Sakon Nakhon Province, Northeastern Thailand. Freshly harvested biomass samples were air-dried in two stages for 1 day each, followed by oven-drying at 80 °C for 3 h. Initially, *Wolffia globosa* was calcined at 350 °C for 190 min in ambient air. The resulting biochar was crushed and sieved. Subsequently, the temperature was gradually increased at a rate of 10 °C min^–1^ from ambient temperature to the set carbonization temperatures. Five carbonization temperatures were used, 900 °C (W900), 1000 °C (W1000), 1100 °C (W1100), 1200 °C (W1200), and 1300 °C (W1300), under an argon (Ar) atmosphere with a constant gas flow rate of 100 mL min^–1^. The samples were heated at a rate of 10 °C min^–1^ to the target temperatures, held for 2 h, and then naturally cooled to room temperature under continuous Ar flow. The substances obtained at these temperatures were utilized as precursors for the production of graphite-like carbon.

### 2.2 Characterization

The graphite-like carbon samples, prepared from *Wolffia globosa*, were comprehensively characterized to investigate their structural, chemical, and electronic properties. Thermogravimetric analysis (TGA) was performed using a TGA 2 thermogravimetric analyzer (Mettler Toledo, Switzerland). Approximately 240 mg of dried *Wolffia globosa* powder was placed in an alumina crucible and heated from 25 to 1000 °C at a heating rate of 10 °C/min under a nitrogen atmosphere with a flow rate of 20 mL/min. The analysis was conducted to evaluate the thermal stability and thermal decomposition behavior of the sample. X-ray diffraction (XRD) patterns were recorded using a Bruker D2 PHASER diffractometer equipped with Cu Kα radiation (λ = 1.54184 Å). Fourier-transform infrared spectroscopy (FTIR, PerkinElmer, USA) was employed to identify the characteristic functional groups. Raman spectroscopy (HORIBA, XploRA PLUS, France, 532 nm laser) was utilized to probe the graphitic structure and degree of disorder of the carbon framework. X-ray photoelectron spectroscopy (XPS, KRATOS Analytical, AXIS Supra+) was used to analyze the surface elemental composition and chemical bonding. The morphology and surface topology of the carbon samples were examined using scanning electron microscopy (SEM, CIQTEK SEM3200, CIQTEK Co., Ltd., China). To further elucidate the internal nanostructure, transmission electron microscopy (TEM, Jeol, JEM-2100Plus, Germany) was employed, providing high-resolution images of layered structures, porosity, and nanoscale defects within the carbon framework. Brunauer–Emmett–Teller (BET, Altamira Instruments, TOP-200, USA) analysis was conducted to assess the morphological properties. Ultraviolet photoelectron spectroscopy (UPS) was performed to investigate the electronic structure of the carbon-based materials, including the work function and valence band maximum (VBM). The measurements were conducted at beamline BL3.2Ua of the Synchrotron Light Research Institute (SLRI, Thailand) using a VG Scienta R4000 electron analyzer. During measurements, the samples were negatively biased at 9.54 V, and the binding energies were calibrated with respect to the Fermi edge of gold. Linearly polarized photons with an energy of 60 eV and a spot size smaller than 1 × 1 mm^2^ were used, with the analyzer positioned at 55° relative to the incident light. The analysis chamber maintained a base pressure of 4 × 10^–8^ Pa, and the total energy resolution was approximately 130 meV at room temperature. No pre-etching or charge-neutralization procedures were applied. Tafel polarization measurements were conducted under dark conditions using a potentiostat (Ossila, UK). A symmetric counter electrode (CE–CE). The potential was scanned from −0.6 to 0.6 V at a scan rate of 20 mV s^–1^. The measurements were performed in an iodide/triiodide (I^–^/I_3_^–^) redox electrolyte. The electrolyte was prepared in acetonitrile and consisted of 0.6 M 1-methyl-3-propylimidazolium iodide (MPI), 0.1 M lithium iodide (LiI), 0.05 M iodine (I_2_), 2.5 mM lithium carbonate (Li_2_CO_3_), and 0.5 M *tert*-butylpyridine (TBP), forming a homogeneous solution.

## 3 Results and Discussion

### 3.1 Carbonization Behavior and Chemical Evolution

The thermogravimetric analysis (TGA) curve of dried *Wolffia globosa* powder is shown in Figure 1. A slight weight loss was observed below 150 °C due to the evaporation of surface-adsorbed moisture and volatile compounds. The major thermal decomposition occurred in the temperature range of 250–350 °C, as indicated by the rapid decrease in weight, corresponding to the degradation of the main organic constituents.^22^ Above 350 °C, the rate of weight loss gradually decreased, suggesting the decomposition of more thermally stable components. Additionally, a slow and continuous mass reduction was observed at temperatures above 700 °C.

**Figure 1:**
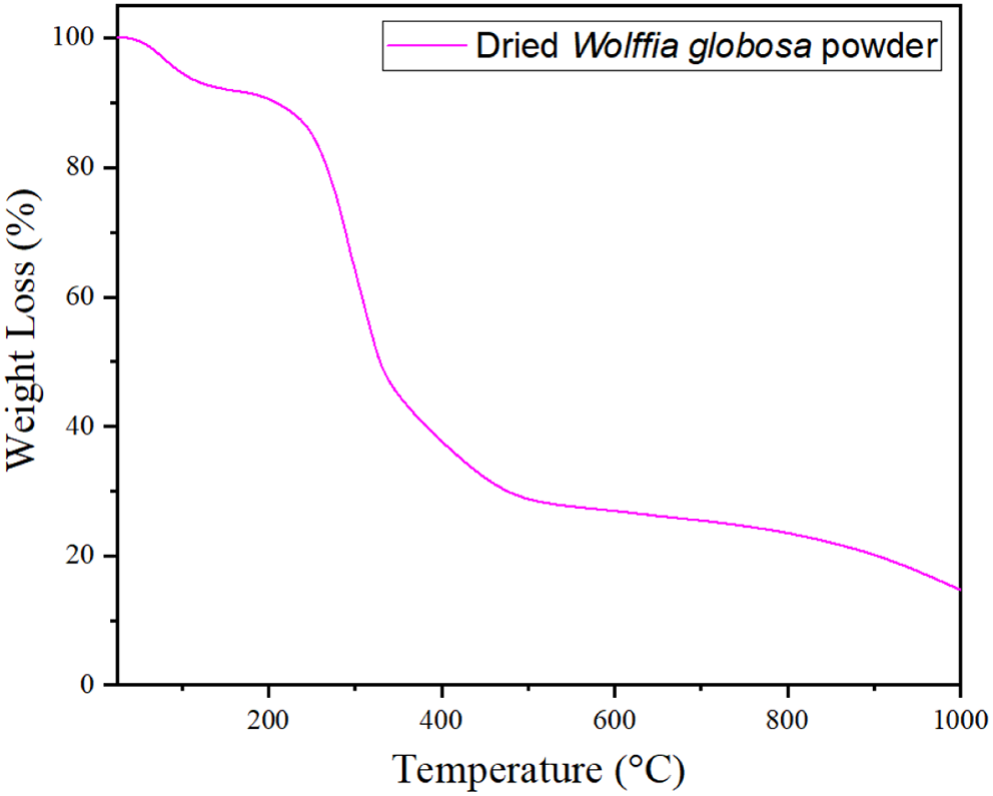
Thermogravimetric analysis curve of dried *Wolffia globosa* powder.

The elemental composition and mass yield of the carbonized samples derived from *Wolffia globosa* are presented in Table 1. All measurements were conducted using four independent samples (n = 4). The dried *Wolffia globosa* powder exhibited low carbon and high oxygen contents of 41.65 ± 0.06% and 48.63 ± 0.26%, respectively, characteristic of oxygen-rich biomass. After carbonization, the carbon content increased progressively from 77.34 ± 0.54% at 900 °C to 90.77 ± 1.12% at 1300 °C, while the hydrogen, nitrogen, and oxygen contents decreased significantly due to devolatilization and thermal decomposition of heteroatom-containing functional groups. The mass yield decreased from 58.05 ± 3.45% to 43.56 ± 3.95% with increasing carbonization temperature, indicating enhanced carbonization at higher temperatures. Notably, W1300 showed the highest carbon content and lowest heteroatom concentrations, suggesting the formation of a highly carbonized structure.

**Table 1:** Elemental Composition (CHNO Analysis) and Mass Yield of Dried *Wolffia globosa*

Sample	CHNO				Mass yield (%)
	C (%)	H (%)	N (%)	O (%)	
Dried *Wolffia globosa* powder	41.65 ± 0.06	6.18 ± 0.31	3.54 ± 0.06	48.63 ± 0.26	-
W900	77.34 ± 0.54	0.99 ± 0.10	1.77 ± 0.02	19.90 ± 0.10	58.05 ± 3.45
W1000	78.34 ± 0.35	0.86 ± 0.04	1.43 ± 0.03	19.37 ± 0.41	53.32 ± 1.99
W1100	78.58 ± 1.31	0.46 ± 0.06	0.50 ± 0.03	20.46 ± 1.40	48.89 ± 1.31
W1200	82.97 ± 2.31	0.08 ± 0.02	0.70 ± 0.02	16.25 ± 2.29	46.11 ± 4.14
W1300	90.77 ± 1.12	0.18 ± 0.19	0.64 ± 0.04	8.41 ± 1.26	43.56 ± 3.95

Fourier transform infrared (FT-IR) spectroscopy was employed to identify the functional groups by analyzing the vibrational modes of the molecules. These results are shown in Figure 2 and Table 2. The FT-IR spectra clearly demonstrate that increasing the carbonization temperature progressively removes the oxygenated functional groups (1052 cm^–1^), including those from the graphite oxide structure. At 900 °C, these functionalities are still abundant, but they gradually diminish and nearly disappear when the temperature reaches 1100–1300 °C. This transformation corresponds to the recovery of a more ordered sp^2^ graphitic framework. Additionally, the stronger C=C stretching band observed at higher temperatures agrees well with the Raman analysis, where a decrease in the I_D_/I_G_ ratio and an enlargement of the crystallite size (L_a_) were detected. These findings show that high-temperature calcination improves graphitization and restores the continuity of the sp^2^ carbon domains.

**Figure 2:**
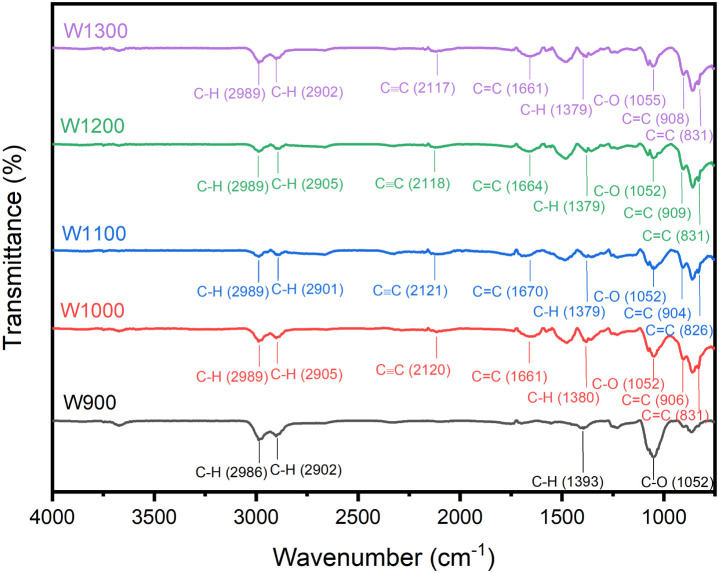
FT-IR spectra of graphite-like carbon from *Wolffia globosa*.

**Table 2:** Functional Groups and Wavenumbers of Graphite-like Carbon from *Wolffia Globosa*

		Group					
Sample		C–H Stretching	C≡C Stretching	C=C Stretching	C–H Bending	C–O Stretching	C=C Bending
Wavenumber (cm^–1^)	W900	2986	-	-	1393	1052	-
		2902					
	W1000	2969	2120	1661	1380	1052	906
		2906					831
	W1100	2989	2121	1670	1379	1052	904
		2901					828
	W1200	2989	2118	1664	1379	1052	909
		2905					831
	W1300	2989	2117	1661	1379	1055	908
		2902					831

### 3.2 Graphitization and Structural Ordering of Graphite-Like Carbon

The X-ray diffraction (XRD) patterns of carbon samples prepared at different carbonization temperatures (W900–W1300) are shown in Figure 3. All samples exhibit two broad diffraction peaks centered at approximately 2θ ≈ 27° and 42°, which are assigned to the (002) and (100) planes of graphitic carbon, respectively.^5,17^ These results are consistent with the TEM observations, which also indicate the formation of partially ordered graphitic domains. The broad nature of these peaks suggests that the carbon materials are predominantly composed of turbostratic carbon rather than highly crystalline graphite. As the carbonization temperature increases, a gradual sharpening and slight increase in the intensity of the (002) peak is observed, indicating an enhanced degree of structural ordering and graphitization. Notably, the W1300 sample exhibits a more pronounced (002) reflection compared to the lower-temperature samples, reflecting improved stacking and alignment of carbon layers along the *c*-axis. These changes in peak characteristics clearly demonstrate a progressive transformation from disordered carbon toward a more graphitic-like structure with increasing thermal treatment.

**Figure 3:**
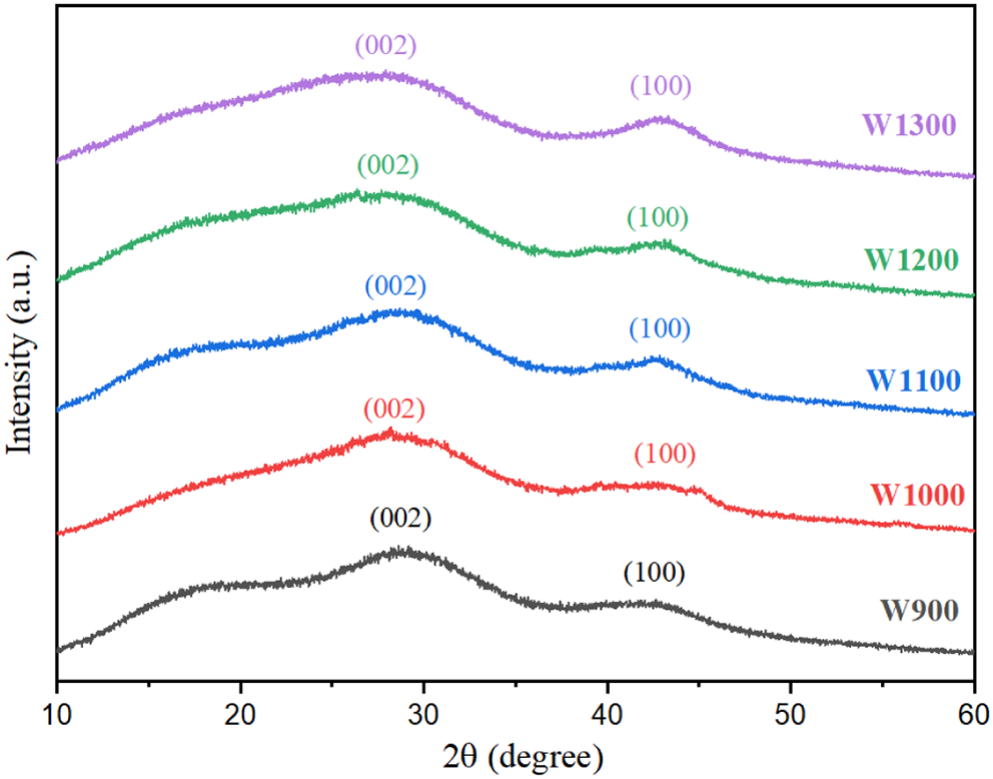
XRD spectra of graphite-like carbon from *Wolffia globosa*.

Raman spectroscopy was employed to investigate the structural characteristics of graphite-like carbon. As shown in Figure 4, the peaks at 1350 cm^–1^ and 1580 cm^–1^ correspond to the D-peaks and the G-peaks, respectively. The D-peak at around 1350 cm^–1^ is associated with structural defects and disorder in the carbon lattice, such as edges, vacancies, and sp^3^-hybridized carbon. In contrast, the G-peak, located at 1580 cm^–1^, is attributed to the in-plane vibration of sp^2^-hybridized carbon atoms, representing the graphitic structure. Samples carbonized at 900, 1000, 1100, 1200, and 1300 °C exhibited I_D_/I_G_ ratios of 1.320, 1.246, 1.113, 1.025, and 1.000, respectively. The decreased I_D_/I_G_ ratio with increasing carbonization temperature suggests enhanced sp^2^ hybridization, which is associated with improved electrical conductivity.^23^ The I_D_/I_G_ values were used to estimate in-plane crystallite size (L_a_) by using the Tuinstra-Koenig relation, which is applicable for a 532 nm excitation source (eq 1).^24^

1 $$L_{a}\left(nm\right)=\left(2.4\times 10^{-10}\right)\lambda_{laser}^{4}{\left(\frac{I_{D}}{I_{G}}\right)}^{-1}$$

**Figure 4:**
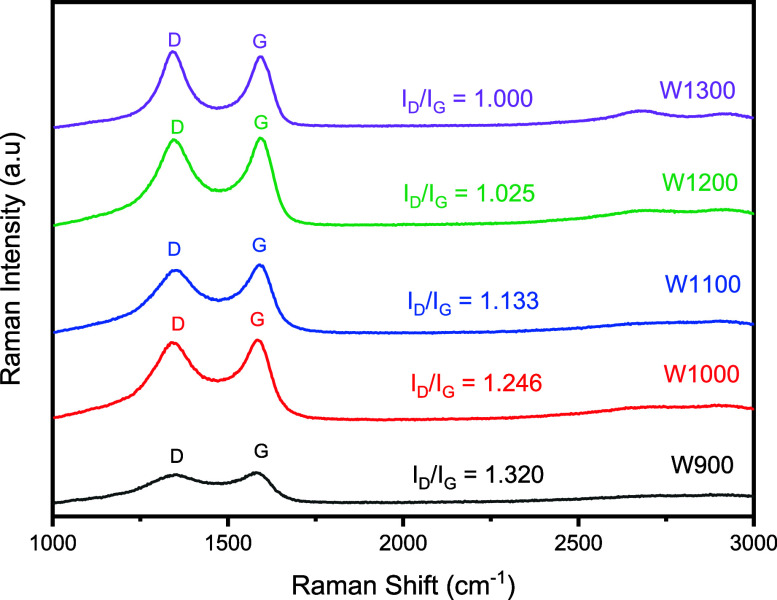
Raman spectra of *Wolffia globosa* graphite-like carbon.

The calculated results show that the crystallite sizes of samples treated at 900, 1000, 1100, 1200, and 1300 °C were 14.56, 15.43, 17.27, 18.76, and 19.22 nm, respectively, as shown in Table 3. The progressive increase in crystallite size can be attributed to the reduction in grain boundary density, which subsequently reduces the internal resistance within the crystallites and thereby improves electrical conductivity.

**Table 3:** Raman Spectroscopic Analysis (I_D_/I_G_) and Crystallite Size of Graphite-like Carbon from*Wolffia globosa*

Sample	I_D_/I_G_ ratio	Crystallite size (nm)
W900	1.320	14.56
W1000	1.246	15.43
W1100	1.133	17.27
W1200	1.025	18.76
W1300	1.000	19.22

X-ray photoelectron spectroscopy (XPS) was employed to investigate the surface elemental composition and chemical bonding states of graphite-like carbon obtained from *Wolffia*, as illustrated in Figure 5a–f. For all samples, two main characteristic peaks corresponding to carbon (C 1s) and oxygen (O 1s) were detected at binding energies of approximately 284.9 and 531.1 eV, respectively.^25^ The high-resolution C 1s spectra of samples carbonized at 900, 1100, and 1300 °C are presented in Figures 5a, c, and e, respectively. Each C 1s spectrum was deconvoluted into four distinct components: sp^2^ C–C bonding (284.8 ± 0.1 eV), sp^3^ C–C bonding (285.6 ± 0.3 eV), C–O functional groups (287.1 ± 0.4 eV), and C=O functional groups (289.1 ± 0.8 eV). These peaks agree with those reported in previous studies.^26,27^ The quantitative analysis of the atomic percentages for these chemical states is summarized in Table 4. A comparison of the relative peak areas of sp^2^ and sp^3^ carbon reveals that samples treated at higher temperatures contain a greater proportion of sp^2^-hybridized carbon compared to sp^3^-hybridized carbon. The calculated sp^3^ /sp^2^ ratios at 900, 1100, and 1300 °C were 0.67, 0.58, and 0.55, respectively. The progressive decrease in this ratio with increasing temperature indicates a structural transformation from sp^3^ to sp^2^ carbon. This observation agrees well with the Raman spectroscopy results, confirming enhanced graphitization and increased structural ordering at elevated carbonization temperatures. The conversion of sp^3^-hybridized carbon into sp^2^ configurations at higher temperatures promotes the development of more graphitic domains within the carbon matrix. The corresponding high-resolution O 1s spectra (Figures 5b, d, and f) were also deconvoluted into three components: C=O bonds (530.7 ± 0.1 eV), C–O groups (around 532.5 eV), and chemisorbed oxygen and/or adsorbed water species (534.2 ± 0.7 eV).^28–30^ All spectra were fitted using Gaussian functions to achieve accurate peak separation and quantitative evaluation.

**Figure 5:**
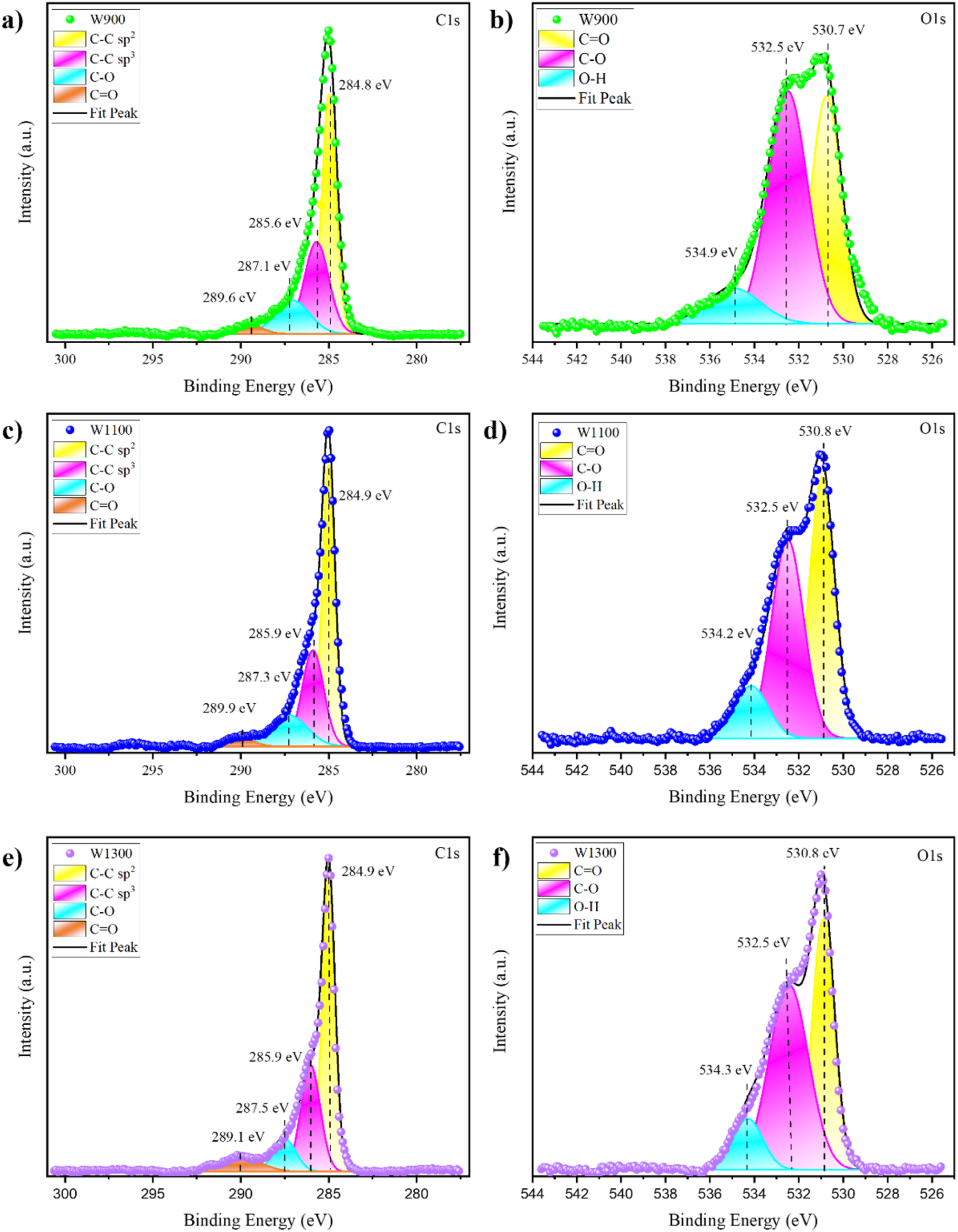
XPS spectra of graphite-like carbon from *Wolffia globosa* (a) C 1s and (b) O 1s of W900; (c) C 1s and (d) O 1s of W1100; and (e) C 1s and (f) O 1s of W1300.

**Table 4:** Summary of the Atomic Percent of Chemical Bonds Characterized by XPS Measurements

	Atomic % (at. %)		
Chemical bonding	900 °C	1100 °C	1300 °C
C–C, sp^2^	48.05	50.75	53.17
C–C, sp^3^	32.03	29.55	29.10
C–O	16.22	16.67	10.32
C=O	3.70	3.03	7.41
sp^3^/sp^2^	0.67	0.58	0.55

### 3.3 Morphology and Textural Characteristics

The morphologies of graphite-like carbon derived from *Wolffia* with different carbonization temperatures are depicted in Figure 6. At lower temperatures, as shown in Figure 6a and b, the structures appear irregular and agglomerated into particles. Figure 6c and d reveal more discernible sheet-like morphologies with increasing temperature, although the surfaces remain relatively rough. At higher temperatures, as presented in Figure 6e–j, thin layered sheets stacked in a more ordered arrangement are observed, with only a few small residual particles attached. These results indicate that higher carbonization temperatures promote the development of thinner, sharper, and more orderly graphitic layers, reflecting an enhanced degree of graphitization. This observation is consistent with the Raman spectroscopy results.

**Figure 6:**
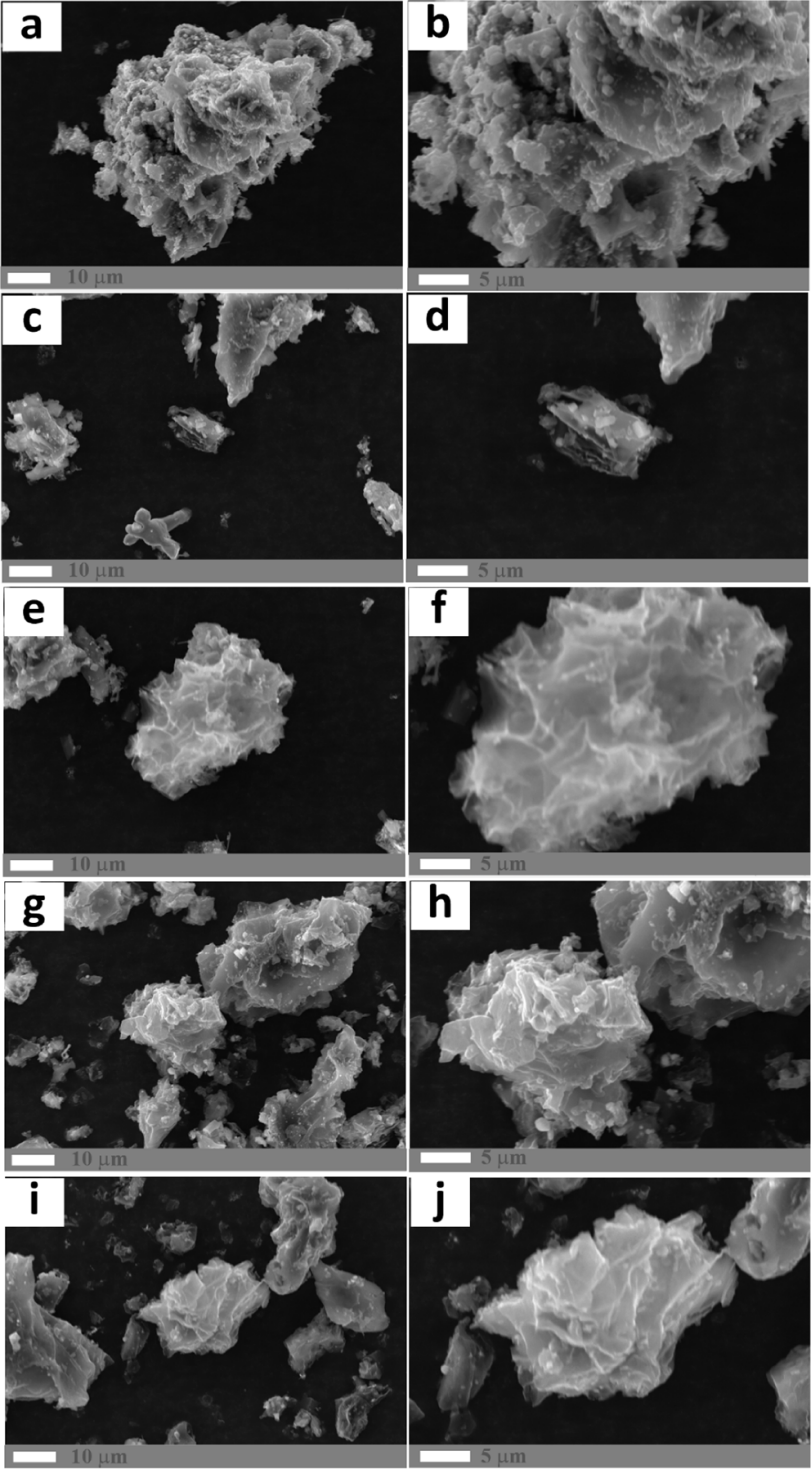
SEM images of graphite-like carbon from *Wolffia globosa* at (a) and (b) 900 °C, (c) and (d) 1000 °C, (e) and (f) 1100 °C, (g) and (h) 1200 °C, and (i) and (j) 1300 °C.

Figures 7(a1–e1) present TEM images of graphite-like carbon derived from *Wolffia*. These figures show the presence of thin, wrinkled, and partially transparent nanosheets that clearly indicate the formation of graphite-layered structures after thermal treatment. TEM and HRTEM images show that the as-prepared carbon consists of both amorphous and graphitic phases, so we refer to it as graphite-like carbon. The atomic-scale structures revealed by HRTEM in Figure 7(a2–e2) display clear lattice fringes indexed to the (002) plane of graphitic carbon,^31,32^ confirming the presence of ordered graphitic regions. The interlayer spacings (d_002_) are 0.329, 0.367, 0.318, 0.304, and 0.324 nm for W900, W1000, W1100, W1200, and W1300, respectively. These values are close to those of graphite, indicating the formation of graphite-like structures. The SAED patterns (Figures 7(a3–e3)) exhibit ring-like diffraction features corresponding to the (002), (100), and (110) reflections of graphitic carbon. As temperature increases, the diffraction rings become more distinct, signaling the growth of graphitic domains and a transition from amorphous-like to semicrystalline carbon. This structural refinement agrees with the HRTEM observations, confirming that elevated annealing temperatures favor long-range order and an enhanced degree of graphitization.

**Figure 7:**
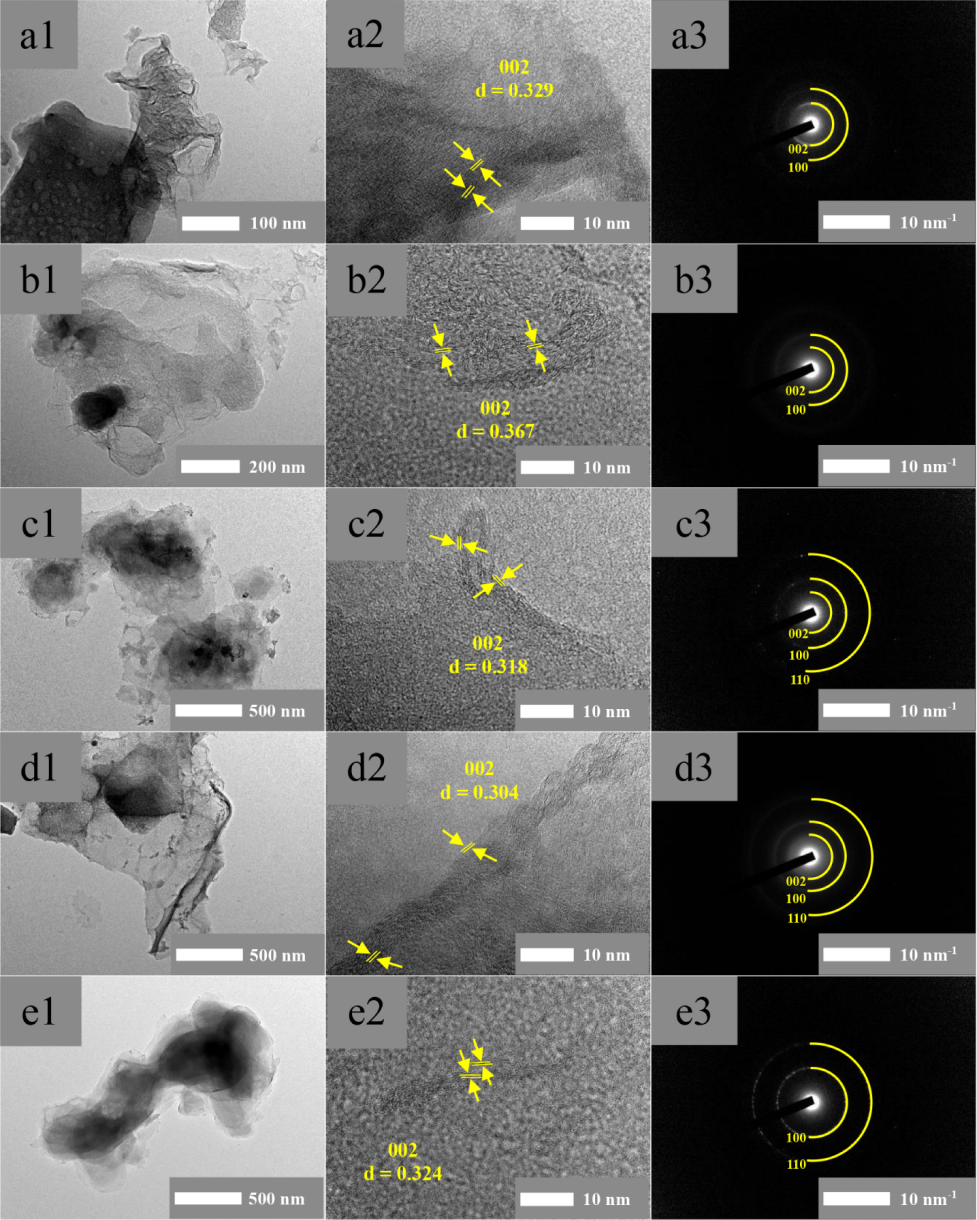
TEM, HRTEM, SAED images of (a1, a2, a3) W900, (b1, b2, b3) W1000, (c1, c2, c3) W1100, (d1, d2, d3) W1200, and (e1, e2, e3) W1300.

Analysis of specific surface area and pore characteristics through nitrogen adsorption–desorption measurements revealed important structural evolution in the W900–W1300 carbon materials. The BET spectra of all examined samples are presented in Figure 8(a–e). According to the IUPAC Technical Report,^33^ all specimens exhibited Type IV isotherms with H4 hysteresis loops, indicating the coexistence of micro–mesoporous structures and slit-shaped pores in these carbonaceous materials. The average pore diameters of W900, W1000, W1100, W1200, and W1300 were 4.85, 6.72, 3.38, 2.85, and 5.42 nm, respectively. A clear correlation was observed between carbonization temperature and BET surface area. The surface area increased markedly with temperature, reaching a maximum in the W1200 sample, which exhibited the highest BET value, 525.6 m^2^/g, suggesting a significant increase in accessible active sites and well-developed pore structures. Such enhancement is beneficial for electrochemical performance, as it promotes improved ion diffusion and charge transfer. The W900 sample exhibited the lowest surface area, 44.42 m^2^/g, indicating limited porosity at lower calcination temperatures. Interestingly, the W1300 sample’s surface area sharply decreased to 87.7 m^2^/g, likely due to partial pore collapse or structural rearrangement at excessively high temperatures. Overall, these findings confirm that the optimal pore development occurs at 1200 °C, where the carbon structure provides the highest surface area and pore volume.

**Figure 8:**
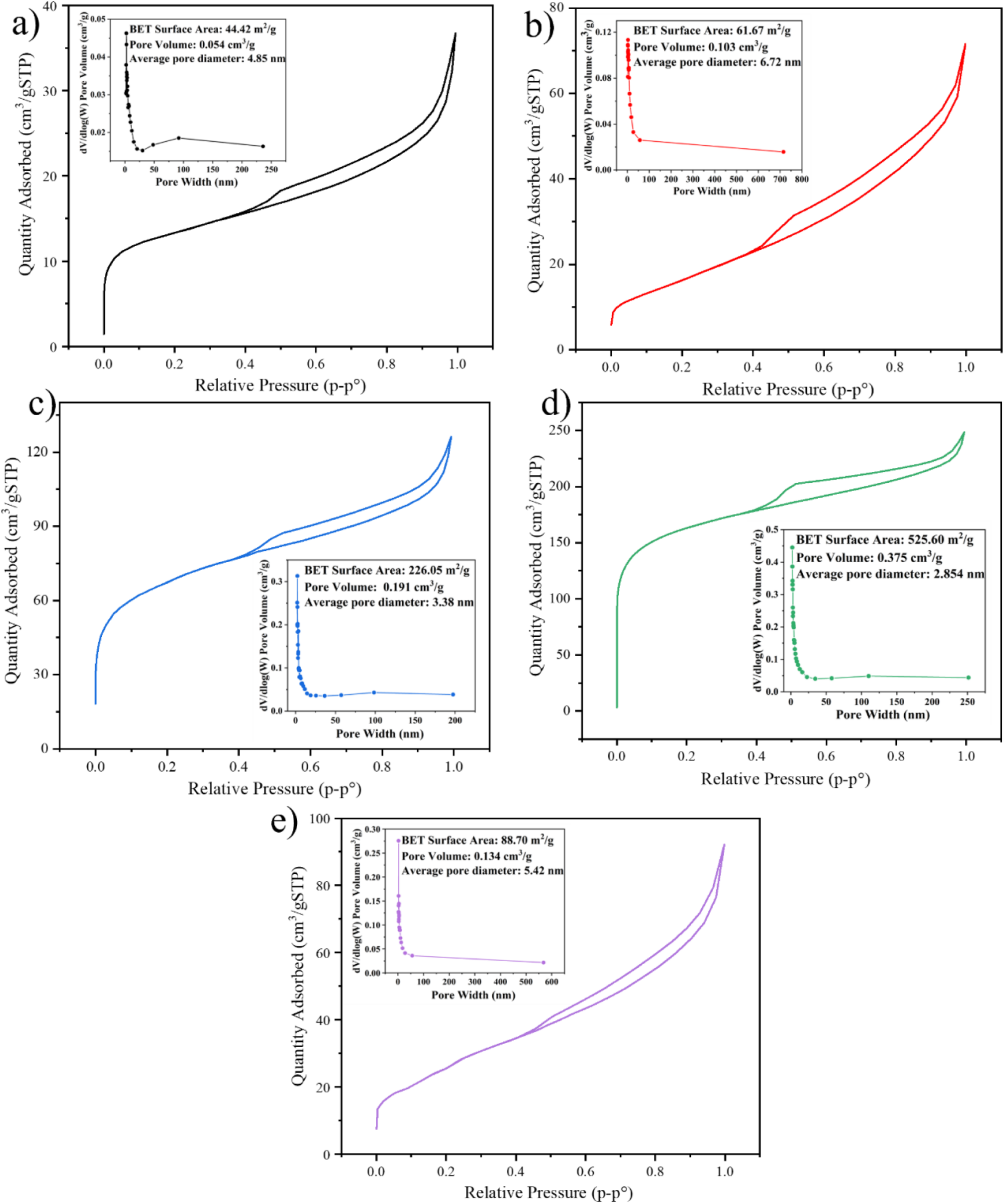
Nitrogen adsorption and desorption isotherms of graphite-like carbon, (a) W900, (b) W1000, (c) W1100, (d) W1200, and (e) W1300.

### 3.4 Work Function and Electronic Property Evolution

Ultraviolet photoelectron spectroscopy (UPS) is an analytical technique used to investigate the electronic structure of materials, particularly the valence states and work function. In this method, the sample surface is irradiated with ultraviolet photons of known energy, causing electrons to be emitted via the photoelectric effect. By measuring the kinetic energy distribution of these emitted electrons, UPS provides detailed insights into the density of states near the Fermi level, the ionization potential, and the work function of the material. In UPS analysis, several key electronic parameters can be extracted to describe the energy-level structure of the material, as illustrated in Figure 9a. The Fermi level (E_F_) is used as the reference energy and corresponds to the highest occupied electronic state at equilibrium, playing an important role in charge transport across interfaces. The vacuum level (E_VAC_) represents the energy of an electron outside the material surface and defines the upper energy limit for electron emission. The work function (WF) is defined as the energy difference between E_VAC_ and E_F_, indicating the minimum energy required to remove an electron from the material surface. The energy gap (E_G_), defined as the difference between the valence band maximum and the conduction band minimum, governs the electronic and optical properties of the material. The ionization energy (IE) corresponds to the energy required to extract an electron from the valence band maximum to the vacuum level, while the electron affinity (EA) is defined as the energy difference between the vacuum level and the conduction band minimum in Figure 9b. This can be used to determine the work function according to eq 2:

2 $$\mathrm{W.F}{.=\mathrm{hv ‐E}}_{\mathrm{cutoff}}$$

where the photon energy (hν) is 60 eV. Figure 10a–c show that the work functions of W900, W1100, and W1300 are 2.66, 3.86, and 4.13 eV, respectively, indicating a clear increase with temperature. This result indicates that the work function of carbon can be effectively tuned through thermal processing. For many applications, such as perovskite solar cells, dye-sensitized solar cells, organic photovoltaics, and supercapacitors, precise control of the carbon work function is essential to achieve optimal energy level alignment. Therefore, this work demonstrates an alternative strategy for tailoring the electronic properties of carbon materials.

**Figure 9:**
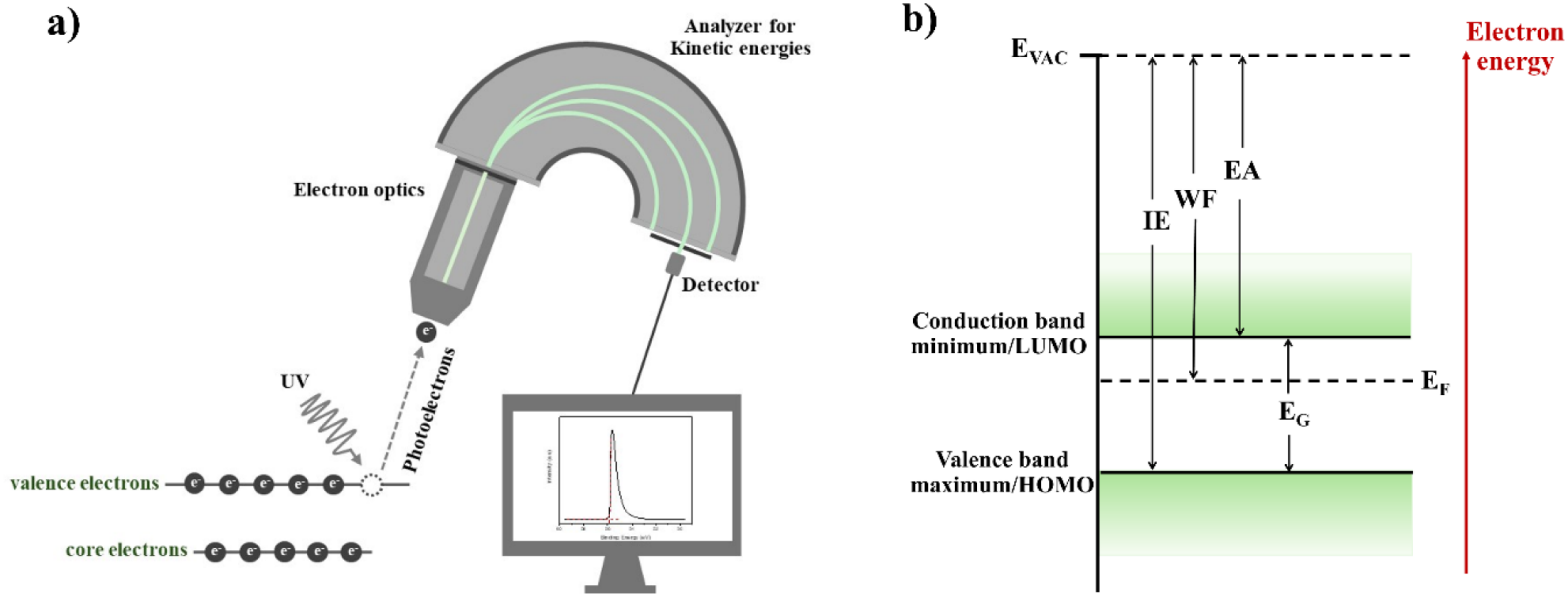
a) Illustration of the fundamental principle of the UPS process, b) Energy level diagram.

**Figure 10:**
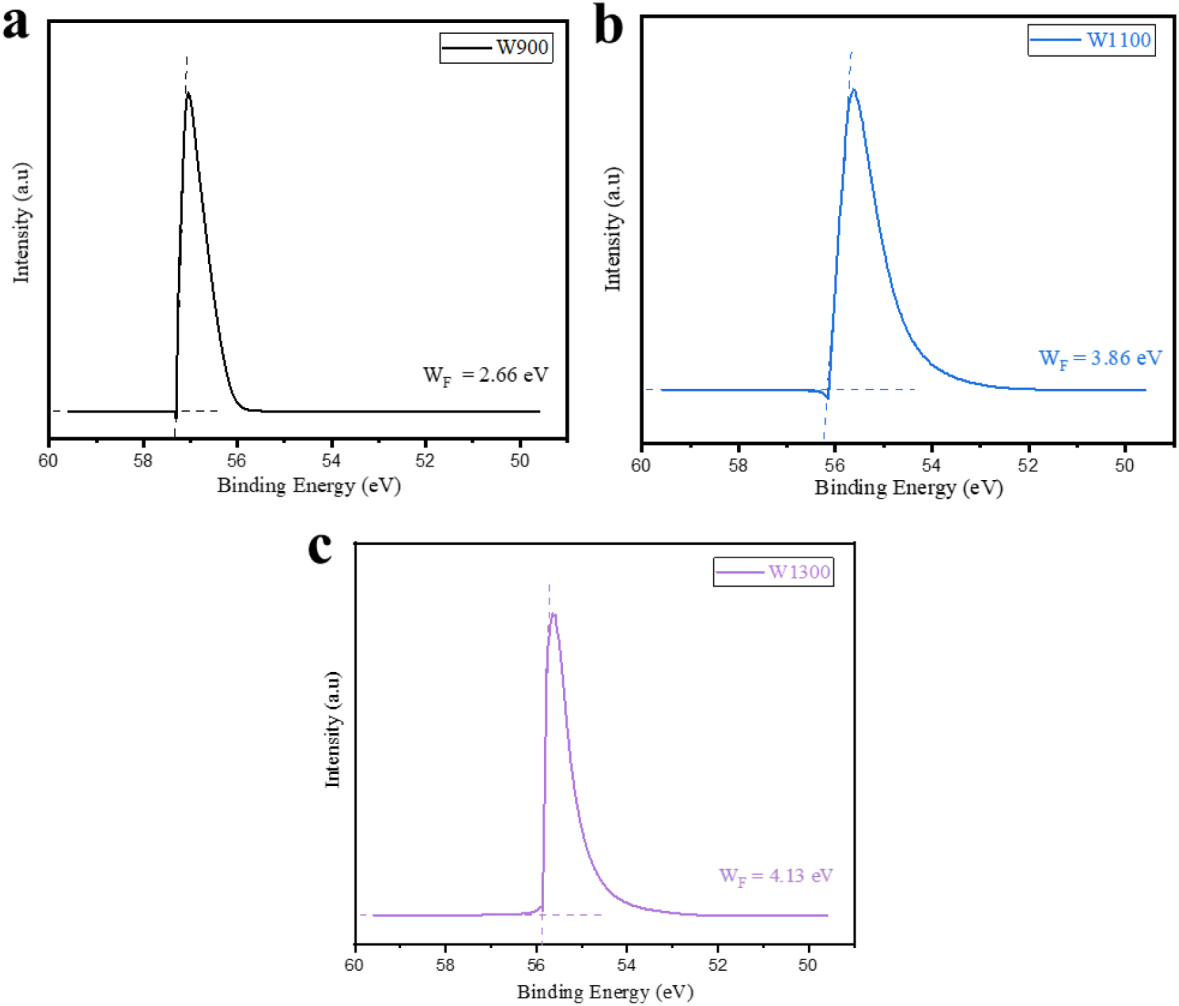
UPS spectra of graphite-like carbon from *Wolffia* at (a) 900 °C, (b) 1100 °C, and (c) 1300 °C.

Thermal defunctionalization (removal of hydroxyl, epoxide, and carboxyl groups) eliminates chemically bonded oxygen that disrupts π-conjugation. This process restores carbon–carbon bonding continuity and generates reactive carbon sites, thereby facilitating lattice reconstruction toward sp^2^ configurations.^34^ With increasing temperature, metastable sp^3^ carbon becomes energetically unfavorable and rearranges into sp^2^-bonded structures. The formation of these initial sp^2^ clusters serves as nucleation centers for extended aromatic domains. The subsequent growth, alignment, and stacking of sp^2^ domains reduce the overall free energy of the system. This ordering process suppresses residual sp^3^ configurations and limits refunctionalization by oxygen, ultimately stabilizing a highly conjugated graphitic framework.^35,36^ Overall, these processes operate synergistically, driving the system from a defect-rich, oxygen-functionalized structure toward a thermodynamically stable, ordered sp^2^ carbon network. Oxygen-containing functional groups introduce local dipoles that can lower or perturb the surface potential depending on their orientation and electronegativity. Their progressive removal reduces dipole-induced screening and leads to a more intrinsic graphitic surface, resulting in an upward shift of the vacuum level relative to the Fermi level.^37^ The transition from sp^3^-dominated (localized, wide bandgap) to sp^2^-dominated (delocalized π-band) carbon fundamentally alters the electronic structure. As extended π-conjugation develops, defect-induced localized states near the Fermi level diminish, and a continuous π-band emerges. This evolution stabilizes the Fermi level at a lower energy relative to the vacuum level, thereby increasing the work function. Graphitization enhances π-electron delocalization and reduces structural disorder. This leads to a narrower distribution of electronic states and a more well-defined electronic structure characteristic of graphitic carbon. Consequently, the energy required to extract an electron increases, yielding a higher and more stable work function compared to amorphous or highly defective carbon.^35,38^

The electrocatalytic activity of redox reactions was assessed using Tafel polarization. Tafel curves of graphite-like carbon are shown in Figure 11. Two important parameters were calculated. They were the exchange current density (*J*_0_) and the limiting current density (*J*_*lim*_) determined using eqs 3 and 4, respectively.^39,40^

3 $$J_{0}=\frac{RT}{nFR_{ct}}$$

where R is the universal gas constant, T is absolute temperature, n is the number of electrons involved in the reduction of triiodide at the electrode, F is Faraday’s constant, and *R_ct_* is the charge transfer resistance.

$$J_{\lim}=\frac{2nDCF}{l}$$ 4

where D, C, and *l* are the mass diffusivity, the triiodide ion concentration, and the interelectrode distance, respectively.

**Figure 11:**
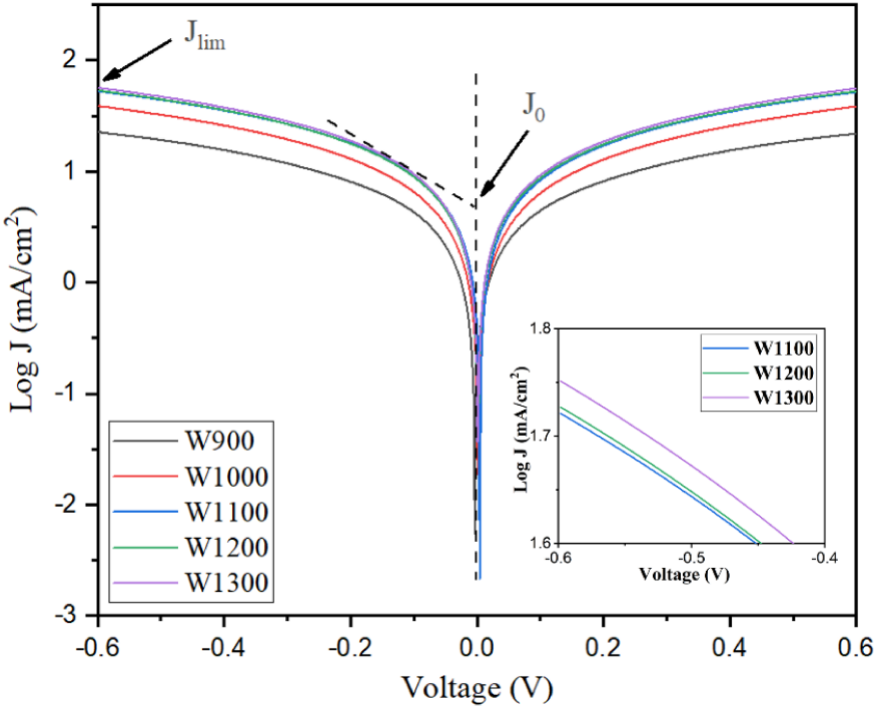
Tafel plots of graphite-like carbon from *Wolffia globosa* mixed PEDOT:PSS films based on FTO glass.

The electrocatalytic performance of the *Wolffia globosa* mixed poly­(3,4-ethylenedioxythiophene):poly­(styrenesulfonate) (PEDOT:PSS) films deposited on fluorine-doped tin oxide (FTO) glass was evaluated by Tafel polarization analysis, and the obtained electrochemical parameters are summarized in Table 5. The W900 sample exhibited the lowest performance, with a J_0_ of 1.337 mA/cm^2^ and a J_lim_ of 0.321 mA/cm^2^, indicating limited electrocatalytic activity. As the preparation temperature increased, the W1000 and W1100 samples showed progressively improved performance, with J_0_ values of 1.588 and 1.706 mA/cm^2^ and J_lim_ values of 0.545 and 0.650 mA/cm^2^, respectively. The W1200 sample further exhibited enhanced activity with a J_0_ of 1.726 mA/cm^2^ and a J_lim_ of 0.654 mA/cm^2^. Finally, the W1300 sample delivered the highest performance, with a J_0_ of 1.746 mA/cm^2^ and a J_lim_ of 0.671 mA/cm^2^, reflecting more efficient charge transfer and improved redox kinetics. The observed enhancement is primarily associated with the evolution of electronic properties, as evidenced by UPS analysis. The UPS results reveal a tunable work function, which may promote more efficient charge-transfer behavior. Furthermore, the improved structural ordering observed from Raman and XRD analyses supports this trend and contributes to enhanced electrical conductivity.

**Table 5:** Summary of Tafel Plot Parameters of Prepared Samples

Sample	J_0_ (mA/cm^2^)	J_lim_ (mA/cm^2^)
W900	1.337	0.321
W1000	1.588	0.545
W1100	1.706	0.650
W1200	1.726	0.654
W1300	1.746	0.671

## 4 Conclusions

This study demonstrates that carbonization temperature plays a crucial role in governing the structure, physical properties, and electronic characteristics of graphite-like carbon derived from *Wolffia globosa*. As the temperature increases, the carbon framework becomes more ordered, with a higher degree of graphitization and larger crystallite size, while the interlayer spacing decreases toward that of graphite. These observations are consistent with Raman, XRD, and TEM analyses and result in improved electrical conductivity and electrochemical behavior. The results currently indicate different optimal samples depending on the characterization technique. The W1300 sample exhibits enhanced electrical conductivity based on the I_D_/I_G_ ratio and crystallite size analysis. The W900 sample shows a lower work function from UPS analysis, making it suitable for solar cell applications. The W1200 sample demonstrates a higher BET surface area, favoring electrochemical applications. Meanwhile, the W1300 sample again exhibits lower charge-transfer resistance from Tafel analysis, indicating superior electrochemical performance. Overall, these findings confirm that *Wolffia globosa* is a sustainable and promising biomass precursor for the production of tunable graphite-like carbon. Precise control of the carbonization temperature is therefore essential for tailoring material properties to specific applications, as different devices require distinct electrode characteristics, such as high surface area, good electrical conductivity, or appropriate work-function alignment.
